# Supplementary material for: Dynamic Organellar Mapping in yeast reveals extensive protein localization changes during ER stress
Source: Nat Commun. 2025 Dec 2;16:10842. doi: 10.1038/s41467-025-66946-8 (PMC12672650; doi:10.1038/s41467-025-66946-8)
Supplement: Supplementary file 1 — Supplementary Information [file 41467_2025_66946_MOESM1_ESM.pdf]

Dynamic Organellar Mapping in yeast reveals extensive protein localization changes during ER stress

Anna Platzek, Klára Odehnalová, Julia P Schessner, Georg H H Borner, Sebastian Schuck

		page
**Figure 1.**	Leakage of lumenal organelle proteins from the ER, mitochondria, the vacuole and the nucleus into the cytosol.	2
**Figure 2.**	Quality assessment of organellar maps of unperturbed yeast.	3
**Figure 3.**	Protein abundance changes upon ER stress.	5
**Figure 4.**	Divergent protein abundance changes upon DTT and tunicamycin treatment.	7
**Figure 5.**	Protein localization changes upon ER stress.	8
**Figure 6.**	Cytosolic redistribution of lumenal ER proteins and reticulon clustering upon ER stress.	10
**Figure 7.**	Redistribution of vacuole towards the ER upon ER stress.	12
**Figure 8.**	Redistribution of plasma membrane and Golgi proteins towards the ER upon ER stress.	13
**Figure 9.**	Redistribution of nucleoporins and importins and disturbed nuclear import upon ER stress.	15
**References**		17
**Source data**		18

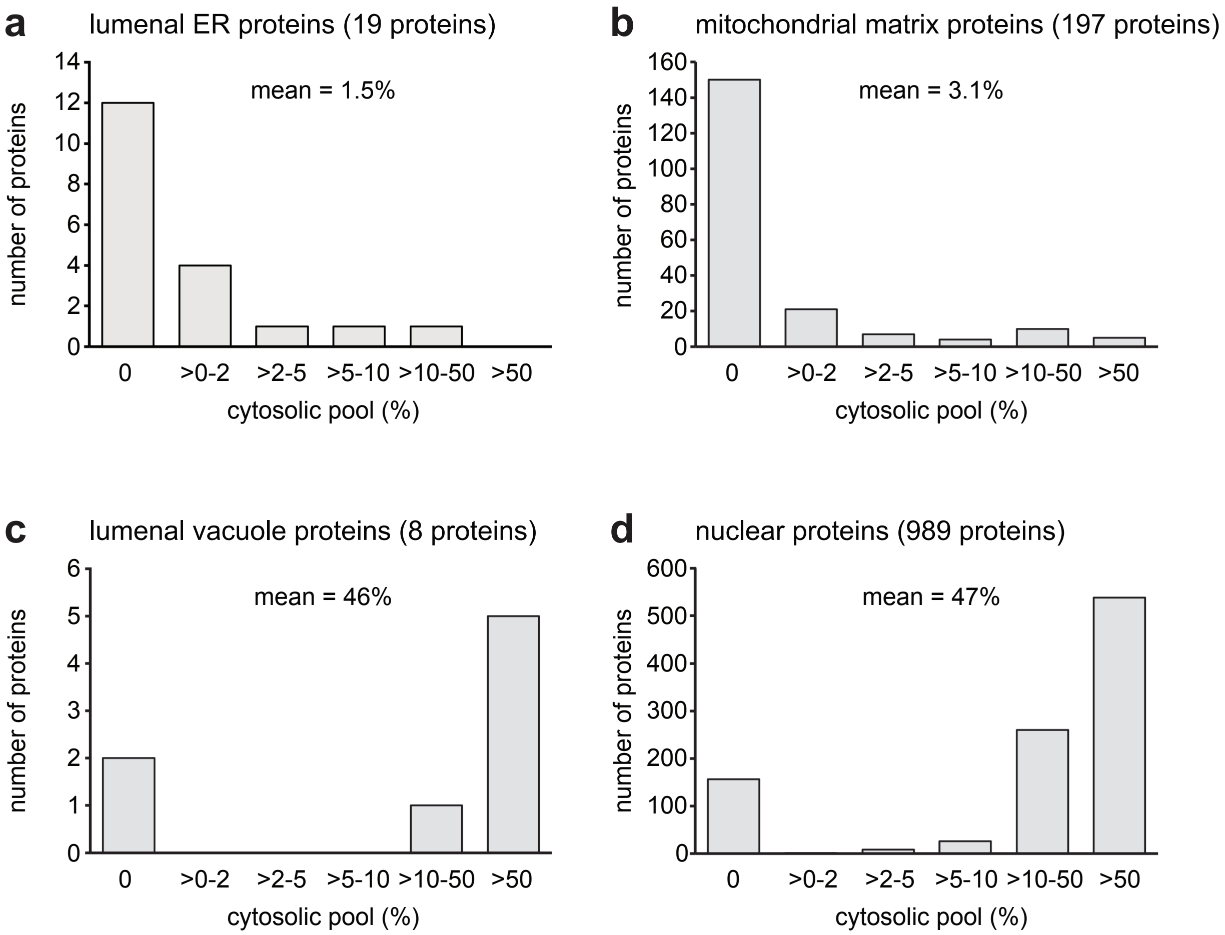

Supplementary Figure 1. Leakage of lumenal organelle proteins from the ER, mitochondria, the vacuole and the nucleus into the cytosol. a Number of lumenal ER proteins with cytosolic pools of 0, >0-2, >2-5, >5-10, >10-50 or >50%. Lumenal proteins were predicted based on the presence of a signal peptide and the absence of a transmembrane domain. Cytosolic pool estimates were derived by dividing protein intensities in the cytosol fraction by the summed intensities in the cytosol and all organelle fractions (Supplementary Data 1b). The mean cytosolic pool of lumenal ER proteins was 1.5%, indicating that ER membranes resealed quickly after cell lysis. **b** As in (a) but for soluble mitochondrial proteins that contain transit peptides, do not have transmembrane domains and have not been identified as intermembrane space proteins[1]. These proteins are predicted to localize to the mitochondrial matrix. Their mean cytosolic pool is 3.1%, indicating that mitochondria remain intact during cell lysis or reseal quickly. **c** As in (a) but for lumenal vacuole proteins. Their mean cytosolic pool is 46%, indicating that many vacuoles are broken during cell lysis, leading to extensive leakage of lumenal proteins. **d** As in (a) but for nuclear proteins. Their mean cytosolic pool is 47%, indicating that nuclei are severely damaged during cell lysis and many nuclear proteins are liberated. Note that the nuclear proteins do not include the nuclear envelope proteins. Source data are provided as a Source Data file.

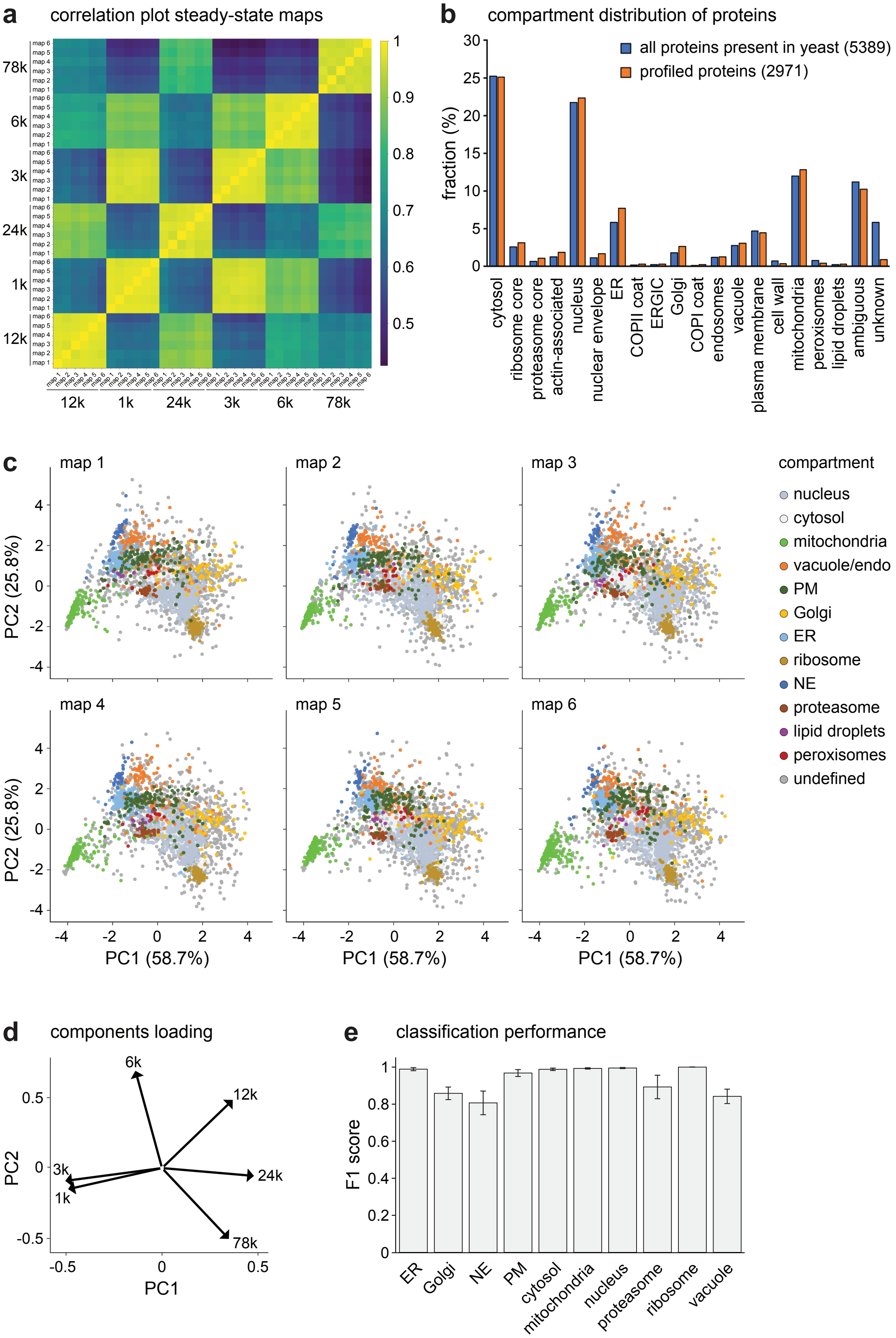

a
correlation plot steady-state maps
78k
6k
3k
24k
1k
12k
map 1
map 2
map 3
map 4
map 5
map 6
1
0.9
0.8
0.7
0.6
0.5
b
compartment distribution of proteins
all proteins present in yeast (5389)
profiled proteins (2971)
fraction (%)
30
25
20
15
10
5
0
cytosol
ribosome core
proteasome core
actin-associated
nucleus
nuclear envelope
ER
COPII coat
ERGIC
Golgi
COPI coat
endosomes
vacuole
plasma membrane
cell wall
mitochondria
peroxisomes
lipid droplets
ambiguous
unknown
c
map 1
map 2
map 3
map 4
map 5
map 6
PC2 (25.8%)
PC1 (58.7%)
compartment
nucleus
cytosol
mitochondria
vacuole/endo
PM
Golgi
ER
ribosome
NE
proteasome
lipid droplets
peroxisomes
undefined
d
components loading
6k
12k
3k
1k
24k
78k
PC2
PC1
0.5
0
-0.5
e
classification performance
F1 score
1
0.8
0.6
0.4
0.2
0
ER
Golgi
NE
PM
cytosol
mitochondria
nucleus
proteasome
ribosome
vacuole

Supplementary Figure 2. Quality assessment of organellar maps of unperturbed yeast. a Heatmap correlation plot of steady-state maps. Plotted are the pairwise Pearson correlations of all subcellular fractions and replicates. Fraction replicates have high reproducibility (R > 0.94). **b** Bar graph of compartment distribution of all 5389 proteins in the reference database and of the 2971 profiled proteins. Compartment assignments for the profiled proteins were taken from the reference database. The compartment distributions are nearly identical, except that the profiled proteins contain fewer proteins with unknown localization. The profiled proteins are biased towards high abundance whereas proteins with unknown localization are biased towards low abundance, explaining why they are underrepresented among the profiled proteins. **c** PCA plots of all six steady-state organellar maps obtained from untreated yeast. Pre-defined compartment markers are colored, all other proteins are classified as 'undefined'. The shared proteins of these maps were used to generate the map shown in Figure 1B. **d** PCA loadings plot for steady-state maps. The plot shows to what extent abundance in each subcellular fraction contributes to the position of a protein in the PCA plot shown in (b). **e** Performance assessment of SVM classifications for steady-state maps. F1 scores indicate classification performance for each compartment, with perfect recall and precision yielding a score of 1. Error bars show standard deviation of repeated subsampling of predictions on the test set. Lipid droplets and peroxisomes had fewer than 20 marker proteins and performance for these comparments was not evaluated. NE, nuclear envelope; PM, plasma membrane. Source data are provided as a Source Data file.

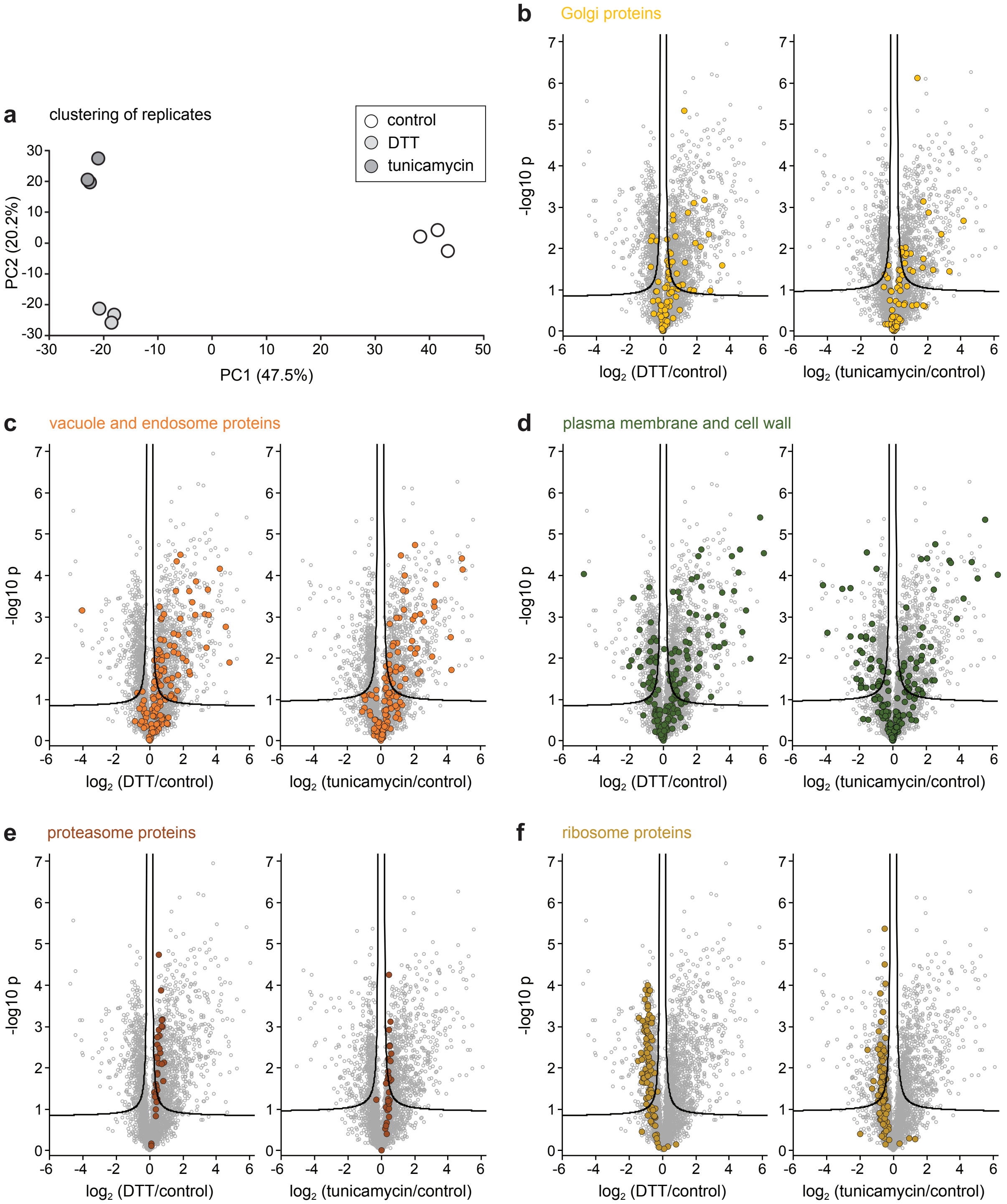
a
clustering of replicates
control
DTT
tunicamycin
PC2 (20.2%)
PC1 (47.5%)
b
Golgi proteins
c
vacuole and endosome proteins
d
plasma membrane and cell wall
e
proteasome proteins
f
ribosome proteins
-log10 p
log2 (DTT/control)
log2 (tunicamycin/control)

Supplementary Figure 3. Protein abundance changes upon ER stress. a PCA plot of all three treatments and replicates based on protein intensities from full proteome samples. The plot shows that replicates cluster tightly within conditions and that all three conditions are clearly resolved. **b** Volcanos plots of full proteomes, showing abundance changes upon DTT or tunicamycin treatment. Plotted are the $\log_2$ fold changes upon treatment. P-values for the significance of changes were calculated with a two-sided t-test (n = 3). Volcano lines indicate 5% false discovery rate cut-offs based on data permutation. Golgi proteins are highlighted. **c** As in (b) but for vacuole and endosome proteins. **d** As in (b) but for plasma membrane and cell wall proteins. **e** As in (b) but for structural proteins of the proteasome. **f** As in (b) but for structural proteins of the ribosome. Source data are provided as a Source Data file.

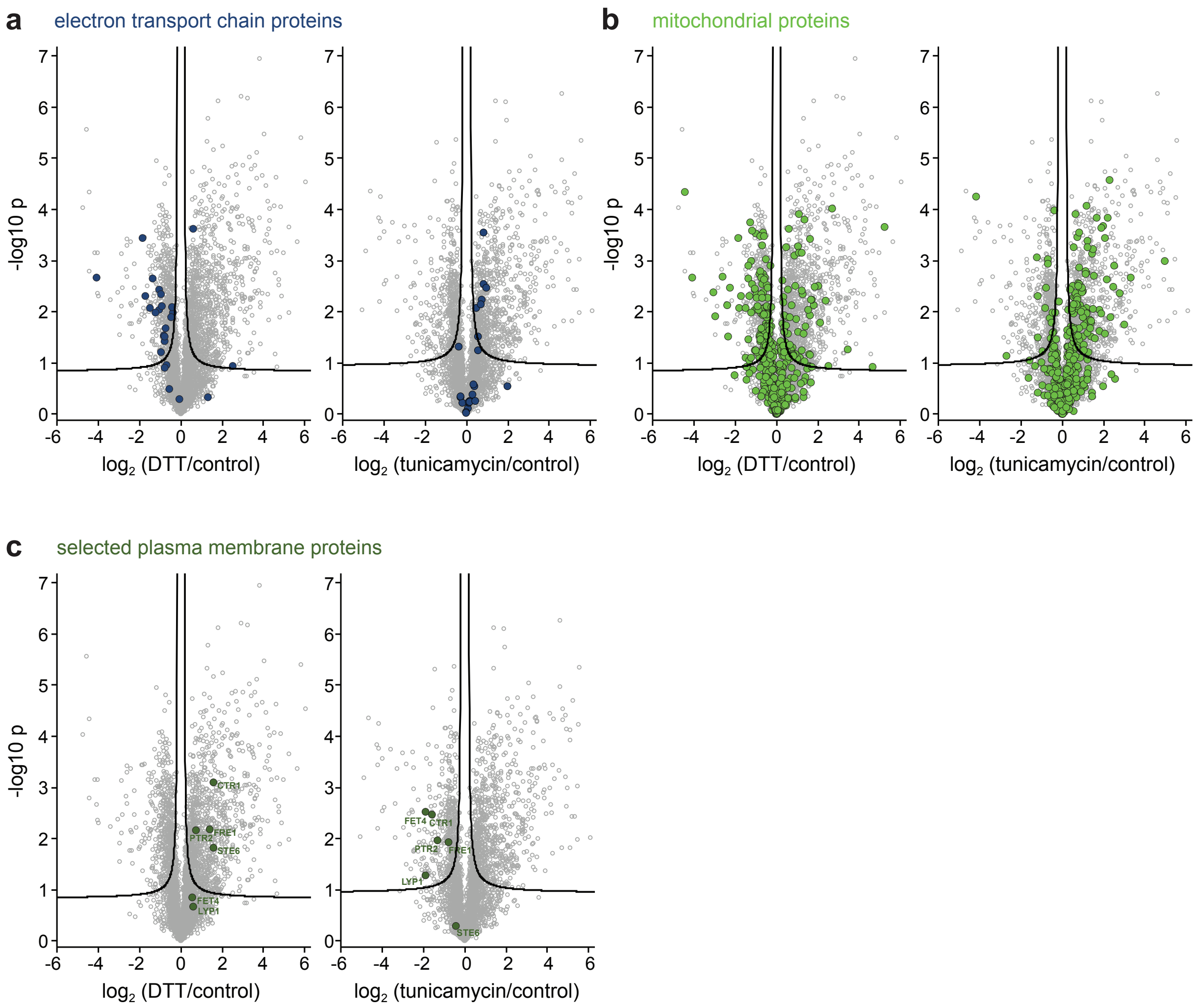

Supplementary Figure 4. Divergent protein abundance changes upon DTT and tunicamycin treatment. a Volcanos plots of full proteomes, showing abundance changes upon DTT or tunicamycin treatment. Plotted are the $\log_2$ fold changes upon treatment. P-values for the significance of changes were calculated with a two-sided t-test (n = 3). Volcano lines indicate 5% false discovery cut-offs based on data permutation. Components of the mitochondrial electron transport chain are highlighted. DTT caused a decrease in the levels of most components whereas tunicamycin caused an increase. **b** As in (a) but mitochondrial proteins are highlighted. DTT tended to cause a downregulation of mitochondrial proteins and tunicamycin an upregulation. **c** As in (a) but selected plasma membrane proteins are highlighted that are upregulated upon DTT treatment and downregulated upon tunicamycin treatment. All of the highlighted proteins are involved in metabolite transport across the plasma membrane. Source data are provided as a Source Data file.

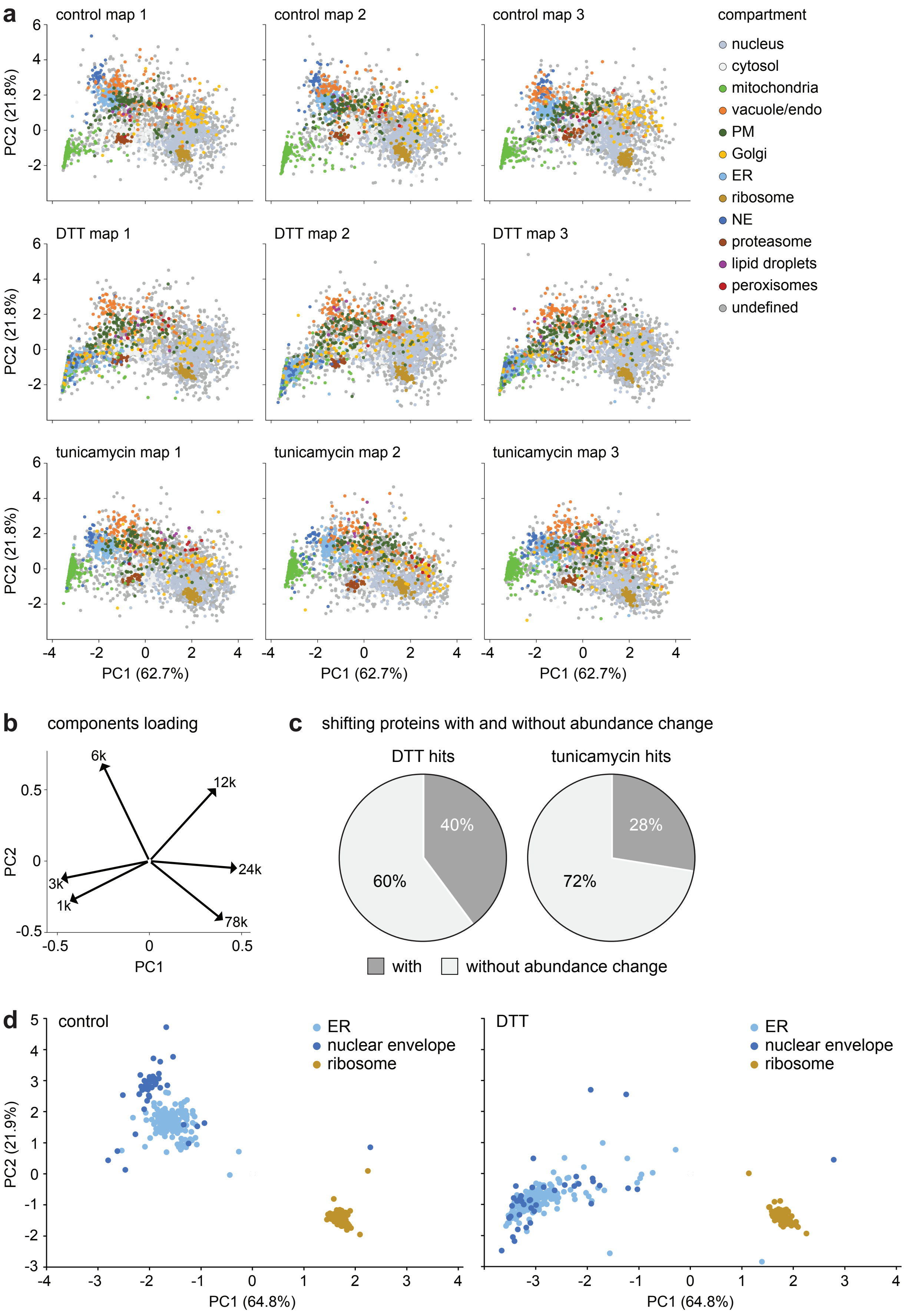
a
control map 1
control map 2
control map 3
DTT map 1
DTT map 2
DTT map 3
tunicamycin map 1
tunicamycin map 2
tunicamycin map 3
PC2 (21.8%)
PC1 (62.7%)
compartment
nucleus
cytosol
mitochondria
vacuole/endo
PM
Golgi
ER
ribosome
NE
proteasome
lipid droplets
peroxisomes
undefined
b
components loading
6k
12k
24k
78k
3k
1k
PC2
PC1
c
shifting proteins with and without abundance change
DTT hits
tunicamycin hits
40%
60%
28%
72%
with
without abundance change
d
control
DTT
ER
nuclear envelope
ribosome
PC2 (21.9%)
PC1 (64.8%)

Supplementary Figure 5. Protein localization changes upon ER stress. a PCA plots of all organellar maps obtained from control, DTT-treated and tunicamycin-treated cells. Pre-defined compartment markers are colored, all other proteins are classified as 'undefined'. The averaged individual profiles of each condition were used to generate the maps shown in Figure 3A. **b** PCA loadings plot. The plot shows to what extent abundance in each subcellular fraction contributes to the position of a protein in the PCA plots in (a). **c** Fraction of shifting proteins with and without accompanying abundance change. The majority of moving proteins do not change in abundance. **d** PCA plots of control and DTT maps highlighting the shifts of the ER and nuclear envelope clusters upon DTT treatment. The ribosome cluster is shown for reference. Source data are provided as a Source Data file.

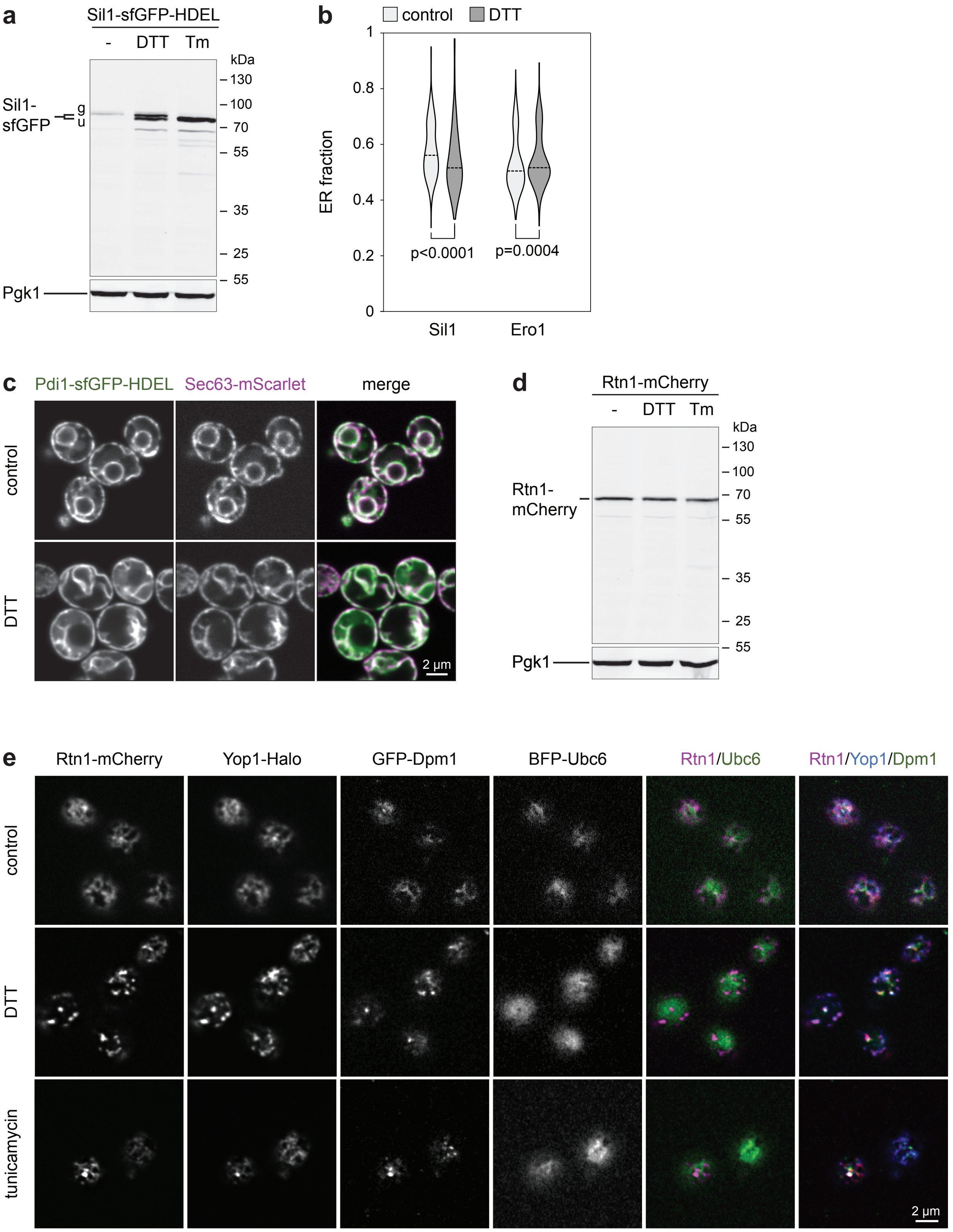
a
Sil1-sfGFP-HDEL
- DTT Tm
kDa
130
100
70
55
35
25
55
Sil1-sfGFP
g
u
Pgk1
b
control DTT
ER fraction
1
0.8
0.6
0.4
0.2
0
p<0.0001
p=0.0004
Sil1
Ero1
c
Pdi1-sfGFP-HDEL
Sec63-mScarlet
merge
control
DTT
2 µm
d
Rtn1-mCherry
- DTT Tm
kDa
130
100
70
55
35
25
55
Rtn1-mCherry
Pgk1
e
Rtn1-mCherry
Yop1-Halo
GFP-Dpm1
BFP-Ubc6
Rtn1/Ubc6
Rtn1/Yop1/Dpm1
control
DTT
tunicamycin
2 µm

Supplementary Figure 6. Cytosolic redistribution of lumenal ER proteins and reticulon clustering upon ER stress. a Western blot of GFP from untreated, DTT-treated and tunicamycin (Tm)-treated cells expressing Sil1-sfGFP-HDEL. g and u mark glycosylated and unglycosylated forms of Sil1-sfGFP-HDEL. Tunicamycin prevents glycosylation of Sil1-sfGFP-HDEL. Besides altered post-translational modification, Sil1-sfGFP-HDEL remained intact during DTT and tunicamycin treatment. **b** Quantification of ER localization of Sil1-sfGFP-HDEL and Ero1-sfGFP in control and DTT-treated cells. The plot shows the ER fraction, which is the ER-localized signal divided by the total cell signal. Dashed lines indicate sample medians. The experiment was performed once, n = 599 cells for Sil1 control, n = 281 cells for Sil1 DTT, n = 717 cells for Ero1 control and n = 459 cells for Ero1 DTT. p-values were calculated with a two-tailed Mann-Whitney U-test. Sil1 shifts away from the ER upon DTT treatment, whereas Ero1 does not. **c** Confocal fluorescence images of mid sections of control and DTT-treated cells expressing the ER marker Sec63-mScarlet and Pdi1-sfGFP-HDEL. **d** Western blot of mCherry from untreated, DTT-treated and tunicamycin (Tm)-treated cells expressing Rtn1-mCherry. **e** Deconvolved confocal fluorescence images of cortical sections of control, DTT-treated and tunicamcin-treated cells expressing the ER marker BFP-Ubc6 along with Rtn1-mCherry, Yop1-Halo and GFP-Dpm1. Rtn1, Yop1 and Dpm1 co-cluster during ER stress, with more extensive clustering in DTT-treated than in tunicamycin-treated cells. Source data are provided as a Source Data file.

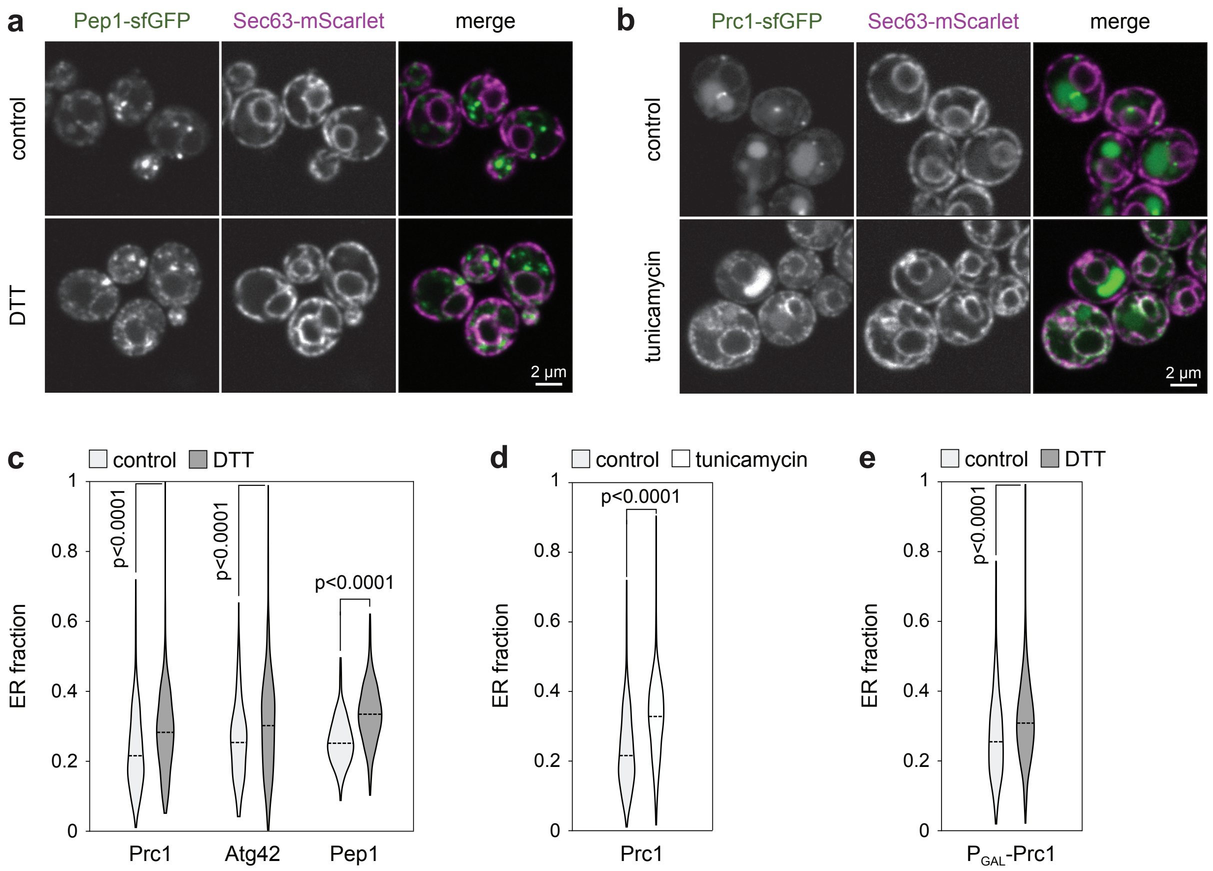

Supplementary Figure 7. Redistribution of vacuole towards the ER upon ER stress. **a** Confocal fluorescence images of mid sections of control and DTT-treated cells expressing the ER marker Sec63-mScarlet and Pep1-sfGFP. **b** Confocal fluorescence images of mid sections of control and tunicamycin-treated cells expressing the ER marker Sec63-mScarlet and the vacuole protein Prc1-sfGFP. **c** Quantification of ER localization of Prc1-sfGFP, Atg42-sfGFP and Pep1-sfGFP in control and DTT-treated cells. The plot shows the ER fraction, which is the ER-localized signal divided by the total cell signal. Dashed lines indicate sample medians. The experiment was performed once, n = 787 cells for Prc1 control, n = 899 cells for Prc1 DTT, n = 284 cells for Atg42 control, n = 1005 cells for Atg42 DTT, n = 397 for Pep1 control and n = 554 for Pep1 DTT. p-values were calculated with a two-tailed Mann-Whitney U-test. All three proteins shift towards the ER upon DTT treatment. **d** As in (c), except for Prc1-sfGFP in control and tunicamycin-treated cells. Note that the data for Prc1 in control cells are the same as in (c). The experiment was performed once, n = 787 cells for Prc1 control and n = 794 cells for Prc1 tunicamycin. Prc1 shifts towards the ER upon tunicamycin treatment. **e** As in (c), except for Prc1-sfGFP that was expressed under an estradiol-inducible system based on the *GAL* promoter (P_{GAL}). Prc1 synthesis was induced in otherwise untreated control cells or during DTT treatment. The experiment was performed once, n = 525 cells for P_{GAL}-Prc1 control and n = 1260 cells for P_{GAL}-Prc1 DTT. Prc1 synthesized during ER stress is more strongly retained in the ER than Prc1 synthesized under control conditions. Source data are provided as a Source Data file.

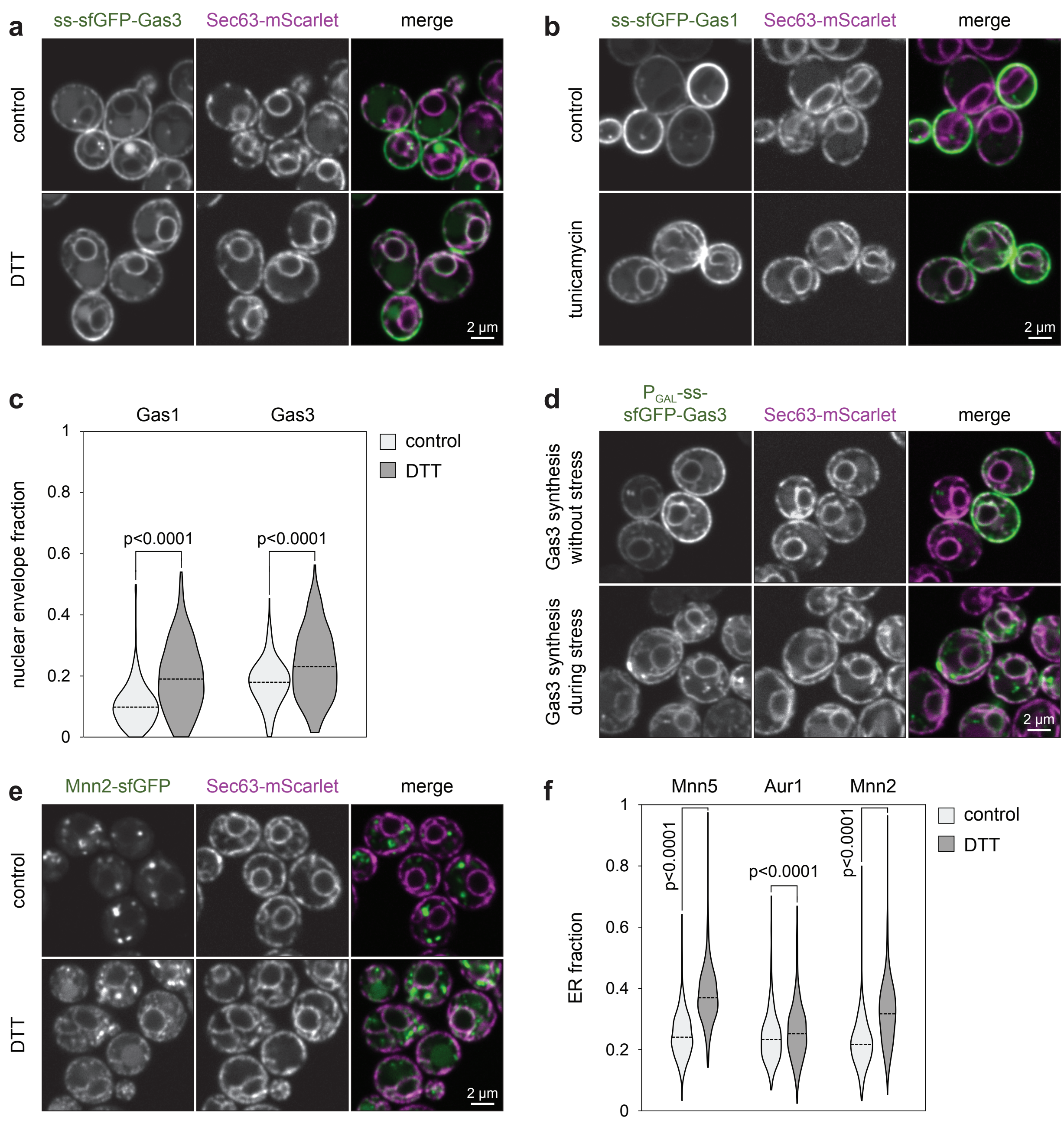

Supplementary Figure 8. Redistribution of plasma membrane and Golgi proteins towards the ER upon ER stress. a Confocal fluorescence images of mid sections of control and DTT-treated cells expressing the ER marker Sec63-mScarlet and ss-sfGFP-Gas3 (ss = signal sequence for ER entry). **b** As in (a) but for control and tunicamycin-treated cells expressing ss-sfGFP-Gas1. **c** Quantification of ER localization of ss-sfGFP-Gas1 and ss-sfGFP-Gas3 in control and DTT-treated cells. Since the peripheral ER and the plasma membrane cannot be resolved by light microscopy, the nuclear envelope signal was used as a proxy for the ER. The plot shows the nuclear envelope fraction, which is the nuclear envelope-localized signal divided by the total cell signal. Dashed lines indicate sample medians. The experiment was performed once, n = 352 cells for Gas1 control, n = 314 cells for Gas1 DTT, n = 350 cells for Gas3 control, n = 160 cells for Gas3

DTT. p-values were calculated with a two-tailed Mann-Whitney U-test. Gas1 and Gas3 shift towards the ER upon DTT treatment. **d** Confocal fluorescence images of cells expressing ss-sfGFP-Gas3 under an estradiol-inducible system based on the *GAL* promoter (P_{GAL}). sfGFP-Gas3 synthesis was induced in otherwise untreated cells (top) or during DTT treatment (bottom). sfGFP-Gas3 synthesized in unstressed cells reaches the plasma membrane (smooth signal along the cell cortext), whereas sfGFP-Gas3 synthesized during ER stress is partially retained in the ER (overlap with Sec63-mScarlet). **e** As in (a) but for Mnn2-sfGFP. **f** Quantification of ER localization of Mnn5-sfGFP, Aur1-sfGFP and Mnn2-sfGFP in control and DTT-treated cells. The plot shows the ER fraction, which is the ER-localized signal divided by the total cell signal. Dashed lines indicate sample medians. The experiment was performed once, n = 838 cells for Mnn5 control, n = 640 cells for Mnn5 DTT, n = 446 cells for Aur1 control, n = 758 cells for Aur1 DTT, n = 846 for Mnn2 control and n = 830 for Mnn2 DTT. p-values were calculated with a two-tailed Mann-Whitney U-test. Mnn5 and Mnn2 shift towards the ER upon DTT treatment, Aur1 does not. The marginal difference between control and DTT-treated cells also reached statistical significance in case of Aur1 due to large sample sizes. Source data are provided as a Source Data file.

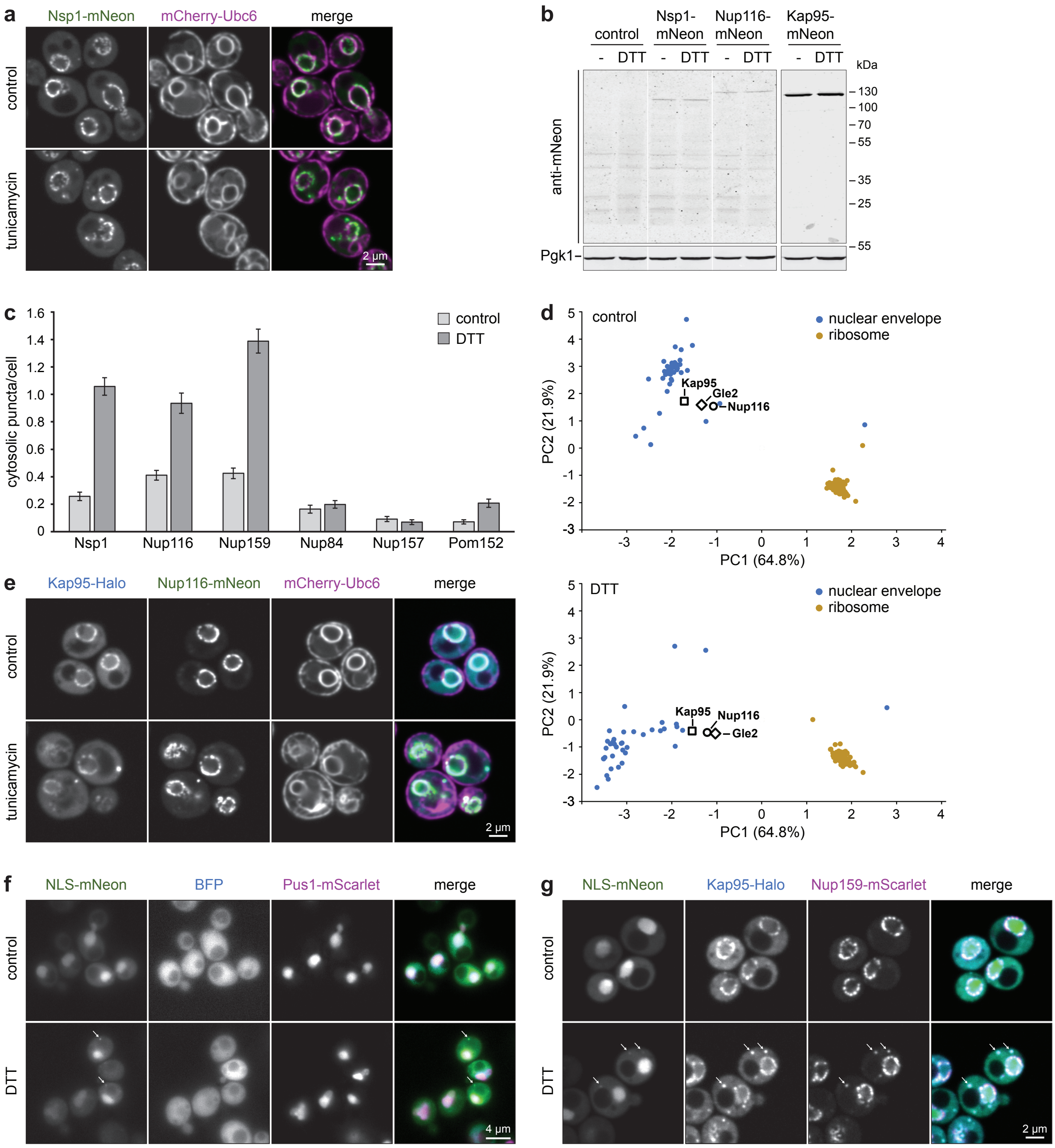

Supplementary Figure 9. Redistribution of nucleoporins and importins and disturbed nuclear import upon ER stress. a Confocal fluorescence images of mid sections of control and tunicamycin-treated cells expressing the ER marker mCherry-Ubc6 and Nsp1-mNeon. **b** Western blot of mNeonGreen from untreated and DTT-treated control, Nsp1-mNeon, Nup116-mNeon and Kap95-mNeon cells. Control cells not expressing an mNeon-tagged protein are shown to demonstrate the specificity of the bands for the mNeon fusion proteins. All fusion proteins remained intact during DTT treatment. **c** Quantification of cytosolic clusters per cell formed by Nsp1, Nup116, Nup159,

Nup84, Nup157 or Pom152 in untreated and DTT-treated cells. The experiment was performed once and the number of cells quantified per condition were, in the order of the bars from left to right, n = 276, 363, 387, 265, 313, 273, 384, 327, 315, 260, 308 and 328. Error bars show the standard error of the mean. **d** PCA plot of organellar maps of control and DTT-treated cells highlighting the shifts of the nucleoporins Nup116 and Gle2 and the importins Kap95. The ribosome cluster is shown for reference. **e** Confocal fluorescence images of mid sections of control and tunicamycin-treated cells expressing the ER marker mCherry-Ubc6 along with the importin Kap95-Halo and the nucleoporin Nup116-mNeon. **f** Confocal fluorescence images of mid sections of control and DTT-treated cells. Cells expressed cytosolic BFP and the nuclear marker Pus1-mScarlet and additonally expressed mNeon fused to a nulcear localization sequence (NLS-mNeon) under the control of an estradiol-inducible promoter system. NLS-mNeon expression was induced for 50 min before imaging. Arrows indicate cytosolic NLS-mNeon puncta in DTT-treated cells. **g** As in (f) except that cells constitutively expressed Nup159-Scarlet and Kap95-Halo, and inducibly expressed NLS-mNeon. Source data are provided as a Source Data file.

Supplementary References

1. Vögtle, F. N. et al. Intermembrane space proteome of yeast mitochondria. *Mol Cell Proteomics* **11**, 1840-1852 (2012).

Source data

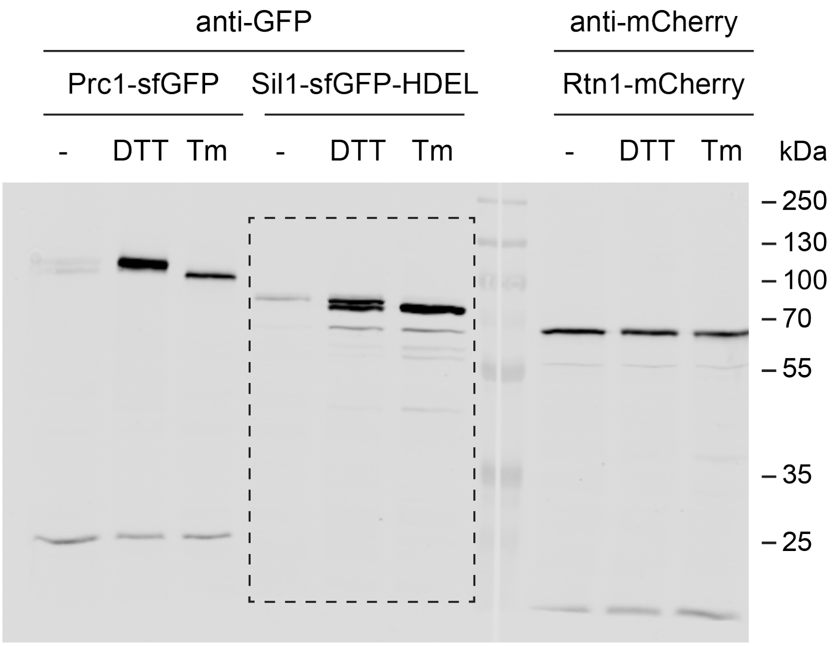

Membrane re-developed with anti-Pgk1 antibody:

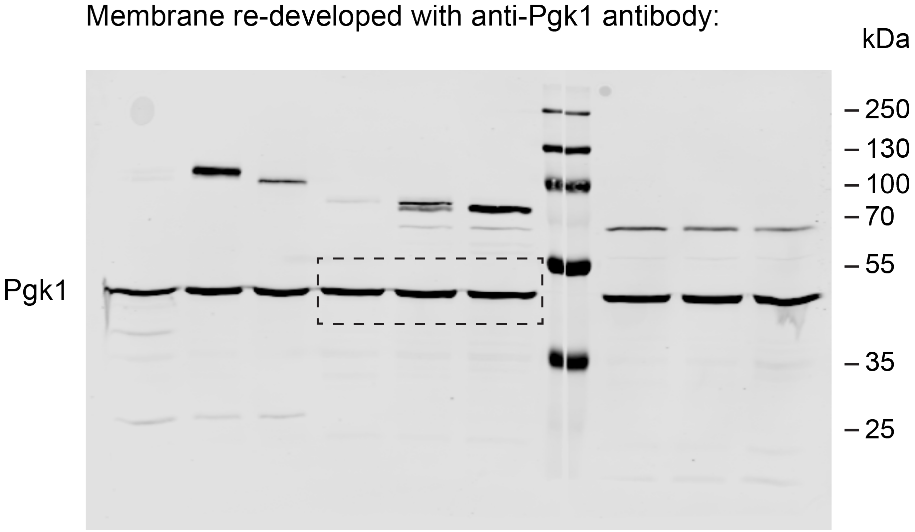

Data for Supplementary Figure 6a. Western blot of GFP from untreated, DTT-treated and tunicamycin (Tm)-treated cells expressing Sil1-sfGFP-HDEL. The cops used for the figure are indicated by dashed lines.

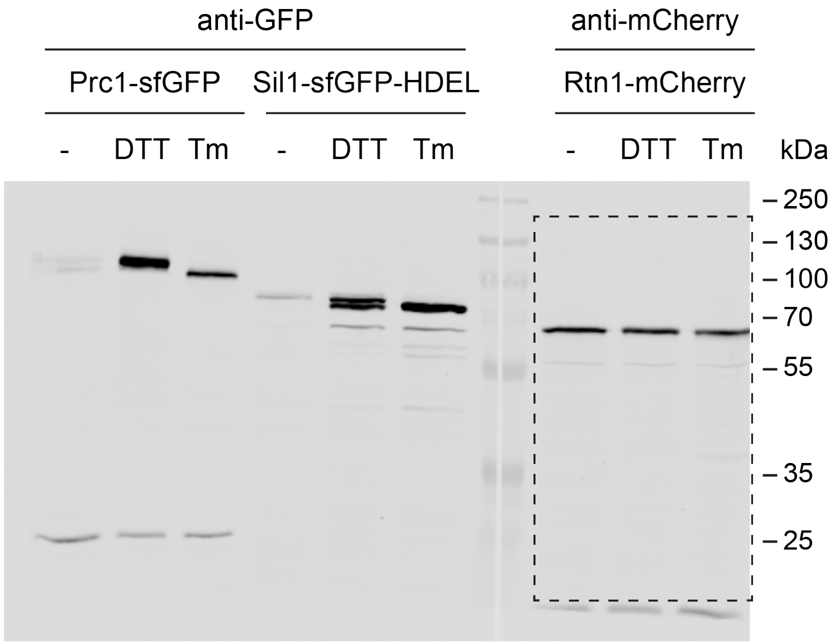

Membrane re-developed with anti-Pgk1 antibody:

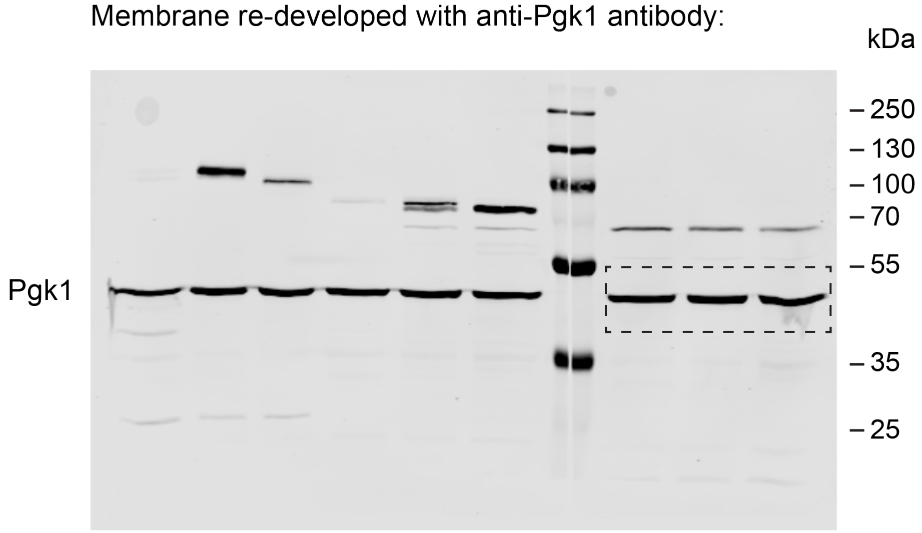

Data for Supplementary Figure 6d. Western blot of mCherry from untreated, DTT-treated and tunicamycin (Tm)-treated cells expressing Rtn1-mCherry. The cops used for the figure are indicated by dashed lines.

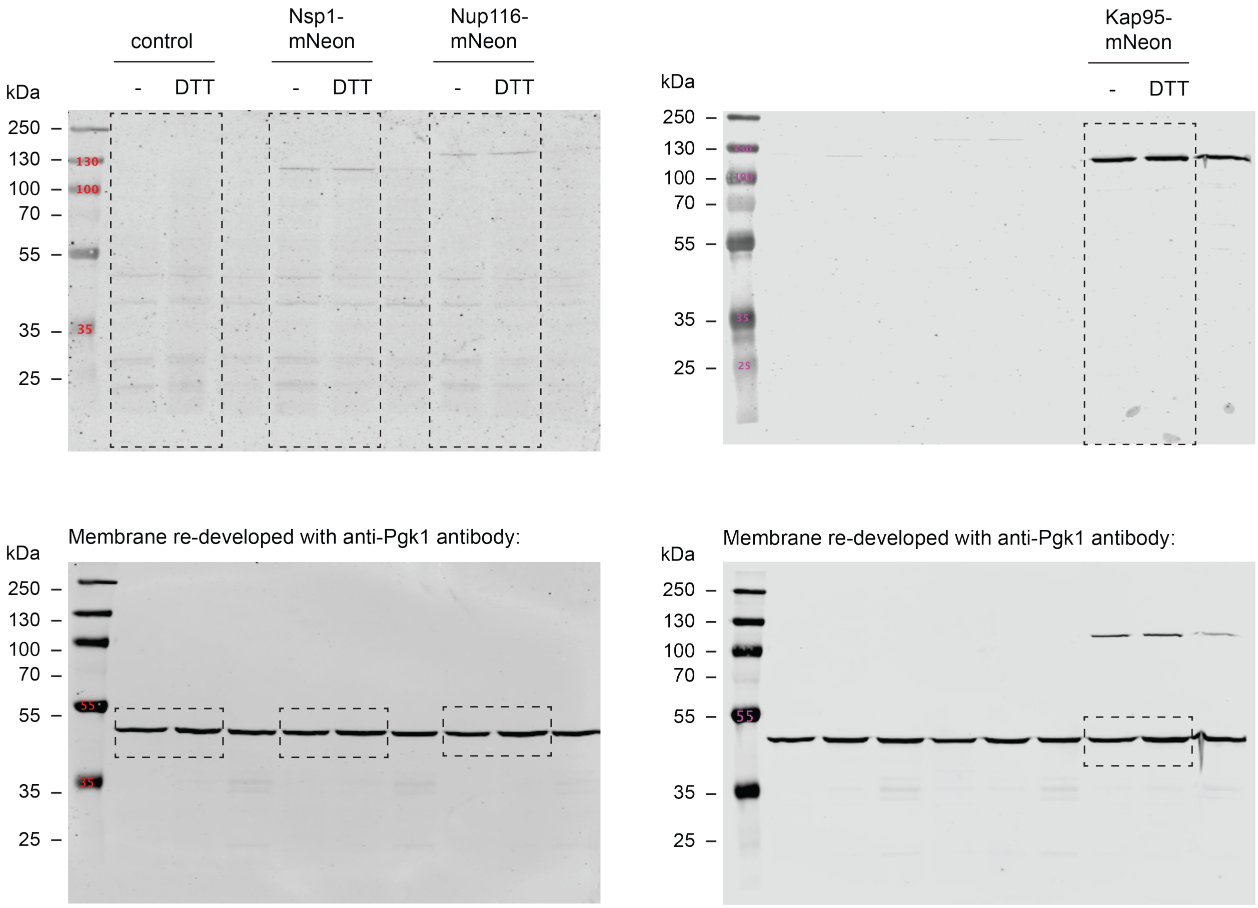

Data for Supplementary Figure 9b. Western blot of mNeonGreen from untreated and DTT-treated control, Nsp1-mNeon, Nup116-mNeon and Kap95-mNeon cells. The cops used for the figure are indicated by dashed lines.
