# Supplementary material for: Dynamic Organellar Mapping in yeast reveals extensive protein localization changes during ER stress
Source: Nat Commun. 2025 Dec 2;16:10842. doi: 10.1038/s41467-025-66946-8 (PMC12672650; doi:10.1038/s41467-025-66946-8)
Supplement: Supplementary file 2 — Reporting Summary [file 41467_2025_66946_MOESM2_ESM.pdf]

nature portfolio

Corresponding author(s): Georg Borner and Sebastian Schuck

Last updated by author(s): Aug 4, 2025

Reporting Summary

Nature Portfolio wishes to improve the reproducibility of the work that we publish. This form provides structure for consistency and transparency in reporting. For further information on Nature Portfolio policies, see our Editorial Policies and the Editorial Policy Checklist.

Statistics

For all statistical analyses, confirm that the following items are present in the figure legend, table legend, main text, or Methods section.

n/a	Confirmed	
☐	☒	The exact sample size (n) for each experimental group/condition, given as a discrete number and unit of measurement
☐	☒	A statement on whether measurements were taken from distinct samples or whether the same sample was measured repeatedly
☐	☒	The statistical test(s) used AND whether they are one- or two-sided<br>*Only common tests should be described solely by name; describe more complex techniques in the Methods section.*
☐	☒	A description of all covariates tested
☐	☒	A description of any assumptions or corrections, such as tests of normality and adjustment for multiple comparisons
☐	☒	A full description of the statistical parameters including central tendency (e.g. means) or other basic estimates (e.g. regression coefficient) AND variation (e.g. standard deviation) or associated estimates of uncertainty (e.g. confidence intervals)
☐	☒	For null hypothesis testing, the test statistic (e.g. F, t, r) with confidence intervals, effect sizes, degrees of freedom and P value noted<br>*Give P values as exact values whenever suitable.*
☒	☐	For Bayesian analysis, information on the choice of priors and Markov chain Monte Carlo settings
☐	☒	For hierarchical and complex designs, identification of the appropriate level for tests and full reporting of outcomes
☐	☒	Estimates of effect sizes (e.g. Cohen's d, Pearson's r), indicating how they were calculated

Our web collection on statistics for biologists contains articles on many of the points above.

Software and code

Policy information about availability of computer code

Data collection	Data collection was done with the help of the following commercial software: Xcalibur s(Thermo Fischer) for the control of the Orbitrap Exploris 480 mass spectrometer, Nikon Nis-Elements Advanced Research version 5.42.03 for the control of the Nikon Ti2 widefield microscope, Nikon Nis-Elements Advanced Research version 5.42.05 for the control of the Nikon Ti2-W1 spinning disk confocal microscope, and LI-COR Image Studio (version 5.2.5).
Data analysis	Data analysis was done with the following commercial or freely available software: Max Quant (version 2.0.1.0), Perseus (version 1.6.2.3 and 2.0.11.0), DOM-ABC, Microsoft Excel, Image J/Fiji (version 1.54p using Java 1.8.0_322), LI-COR Image Studio (version 5.5.4), and Python (version 3.12.7). The custom scripts used for image analysis in Fiji are available at https://gihub.com/SchuckLab/Image_analysis_tools_Platzek and https://doi.org/10.5281/zenodo.17508539 (also see code availability section).

For manuscripts utilizing custom algorithms or software that are central to the research but not yet described in published literature, software must be made available to editors and reviewers. We strongly encourage code deposition in a community repository (e.g. GitHub). See the Nature Portfolio guidelines for submitting code & software for further information.

Data

Policy information about availability of data

All manuscripts must include a data availability statement. This statement should provide the following information, where applicable:

- Accession codes, unique identifiers, or web links for publicly available datasets
- A description of any restrictions on data availability
- For clinical datasets or third party data, please ensure that the statement adheres to our policy

The proteomic datasets generated during the current study are available from the ProteomeXchange Consortium via the PRIDE partner repository with the dataset identifiers PXD061762 and PXD061764. Source Data are provided with this paper.

Research involving human participants, their data, or biological material

Policy information about studies with human participants or human data. See also policy information about sex, gender (identity/presentation), and sexual orientation and race, ethnicity and racism.

Reporting on sex and gender	n/a
Reporting on race, ethnicity, or other socially relevant groupings	n/a
Population characteristics	n/a
Recruitment	n/a
Ethics oversight	n/a

Note that full information on the approval of the study protocol must also be provided in the manuscript.

Field-specific reporting

Please select the one below that is the best fit for your research. If you are not sure, read the appropriate sections before making your selection.

[x] Life sciences [ ] Behavioural & social sciences [ ] Ecological, evolutionary & environmental sciences

For a reference copy of the document with all sections, see nature.com/documents/nr-reporting-summary-flat.pdf

Life sciences study design

All studies must disclose on these points even when the disclosure is negative.

Sample size	No statistical methods were used to predetermine sample size. The number of replicates obtained for dynamic organellar mapping (n = 3 biological replicates of technical duplicates for steady state maps; n = 3 biological replicates for ER stress maps) was chosen based on previous experience with the reproducibility of the technique.
Data exclusions	No data were excluded except during image quantification for Supplementary Figures 6b, 7c, 7d, 7e, 8d and 8f. There, a small number of cells with an ER or nuclear envelope fraction >1 was filtered out during automated image analysis. Such fractions >1 are impossible and reflect inaccurate masking of the cell area.
Replication	The proteomics experiments were done in biological triplicate of technical duplicates (steady state maps) or in biological triplicate (ER stress maps). Microscopy experiments shown in Figures 5c, 5e, 6c, 6d, 7b, 7d, 8b, 8e and Supplementary Figures 6c, 6e, 7a, 7b, 8a, 8b, 8c, 8e, 9a, 9e and 9g were done at least twice to test for reproducibility of the observed localizations. The nuclear import assay shown in Figure 8f and Supplementary Figure 9f was done in biological triplicate. Western blots shown in Figure 6e and Supplementary Figures 6a, 6d and 9a were done once. Image quantification shown in Supplementary Figures 6b, 7c, 7d, 7e, 8d and 8f was done once and cells were quantified from three different fields of view each.
Randomization	The experiments consisted of comparisons of genetically different or differently treated yeast strains, so no randomization was necessary.
Blinding	Blinding was not relevant except for the data underlying Figure 8c, in which two individuals independently assessed microscopy images to determine whether various nucleoporins formed cytosolic puncta. For these assessments, the "Blind Analysis Tool" plugin was used to blind the evaluators to the identity of the imaged strains (see Methods section).

Reporting for specific materials, systems and methods

We require information from authors about some types of materials, experimental systems and methods used in many studies. Here, indicate whether each material, system or method listed is relevant to your study. If you are not sure if a list item applies to your research, read the appropriate section before selecting a response.

Materials & experimental systems

n/a	Involved in the study
	[x] Antibodies
	[x] Eukaryotic cell lines
[x]	Palaeontology and archaeology
[x]	Animals and other organisms
[x]	Clinical data
[x]	Dual use research of concern
[x]	Plants

Methods

n/a	Involved in the study
[x]	ChIP-seq
[x]	Flow cytometry
[x]	MRI-based neuroimaging

Antibodies

Antibodies used	Primary antibodies: mouse anti-GFP (clones 7.1/13.1 from Roche, RRID:AB_390913), mouse anti-mNeonGreen (clone 32F6 from Chromotek, RRID:AB_2827566), mouse anti-mCherry (1C51 from Abcam, RRID:AB_3242120), mouse anti-Pgk1 (clone 22C5D8 from Abcam, RRID:AB_10861977). Secondary antibody: goat anti-mouse Alexa-680 from Invitrogen, RRID:AB_141436.
Validation	The specificity of the anti-GFP, anti-mNeonGreen and anti-mCherry antibodies was established by Western blots comparing cell lysates containing or not containing GFP, mNeonGreen or mCherry. The anti-Pgk1 antibody was not validated. The specificity of the goat anti-mouse secondary antibody was validated by western blotting in the absence of a primary antibody.

Eukaryotic cell lines

Policy information about cell lines and Sex and Gender in Research

Cell line source(s)	Saccharomyces cerevisiae (strain W303) were originally provided by Peter Walter, University of California at San Francisco. All strains used in this study were derived from that parental strain.
Authentication	Genomic modifications were validated by colony PCR and, where applicable, by microscopy.
Mycoplasma contamination	n/a
Commonly misidentified lines (See ICLAC register)	n/a

Plants

Seed stocks	*Report on the source of all seed stocks or other plant material used. If applicable, state the seed stock centre and catalogue number. If plant specimens were collected from the field, describe the collection location, date and sampling procedures.*
Novel plant genotypes	*Describe the methods by which all novel plant genotypes were produced. This includes those generated by transgenic approaches, gene editing, chemical/radiation-based mutagenesis and hybridization. For transgenic lines, describe the transformation method, the number of independent lines analyzed and the generation upon which experiments were performed. For gene-edited lines, describe the editor used, the endogenous sequence targeted for editing, the targeting guide RNA sequence (if applicable) and how the editor was applied.*
Authentication	*Describe any authentication procedures for each seed stock used or novel genotype generated. Describe any experiments used to assess the effect of a mutation and, where applicable, how potential secondary effects (e.g. second site T-DNA insertions, mosiacism, off-target gene editing) were examined.*
