# Supplementary material for: Dynamic Organellar Mapping in yeast reveals extensive protein localization changes during ER stress
Source: Nat Commun. 2025 Dec 2;16:10842. doi: 10.1038/s41467-025-66946-8 (PMC12672650; doi:10.1038/s41467-025-66946-8)
Supplement: Supplementary file 3 — Description of Additional Supplementary Files [file 41467_2025_66946_MOESM3_ESM.pdf]

Supplementary Data 1. Data file containing (A) protein copy numbers, (B) cytosolic pool estimates, (C) SVM predictions based on steady-state maps, (D) a comparison of SVM predictions and the reference database, (E) a comparison of SVM predictions and previous spatial proteomics predictions derived by the LOPIT method, and (F) novel SVM predictions.

Supplementary Data 2. Data file containing (A) the literature-based reference database, (B) the compartment marker list, and (C) topology annotations of ER proteins.

Supplementary Data 3. Interactive database for exploration of yeast steady-state organellar maps and protein abundance data.

Supplementary Data 4. Data file containing (A) a comparison of the full proteome of DTT-treated versus control cells, (B) a comparison of the full proteome of tunicamycin-treated versus control cells, (C) full proteome hits shared by DTT and tunicamycin, (D) unique full proteome hits after DTT or tunicamycin treatment, (E) gene ontology term enrichment analysis of DTT-treated versus control cells, and (F) gene ontology term enrichment analysis of tunicamycin-treated versus control cells.

Supplementary Data 5. Data file containing (A) the organelle shifts analysis, (B) the MR analysis DTT-treated versus control cells, (C) the MR analysis tunicamycin-treated versus control cells, (D) a comparison of full proteome and MR hits, (E) the cytosolic pool analysis

of control, DTT- and tunicamycin-treated cells, (F) SVM prediction based on ER stress maps, and (G) a list of post-ER secretory pathway proteins present in the DTT maps.

Supplementary Data 6. Interactive ER stress maps analysis tool (ESMAT) for exploration of yeast ER stress organellar maps and protein abundance data.

Supplementary Data 7. Data file containing the manual annotation of the 410 DTT hits.

Supplementary Data 8. Data file containing the analysis of all nucleoporins.

Supplementary Data 9. Plasmids used in this study.

Supplementary Data 10. Sequence files of plasmids used in this study.

Supplementary Data 11. Yeast strains used in this study.

Supplementary Data 12. Label-free quantification intensity data and normalized profiles for steady-state maps.

Supplementary Data 13. Label-free quantification intensity data and normalized profiles for ER stress maps.

Supplementary Data 14. Data to recapitulate the analysis with the DOM-ABC software including step-by-step instructions.
