# Supplementary material for: Dynamic Organellar Mapping in yeast reveals extensive protein localization changes during ER stress
Source: Nat Commun. 2025 Dec 2;16:10842. doi: 10.1038/s41467-025-66946-8 (PMC12672650; doi:10.1038/s41467-025-66946-8)
Supplement: Supplementary file 12 — Supplementary Data 9 [file 41467_2025_66946_MOESM12_ESM.pdf]

Supplementary Data 9. Plasmids used in this study. GEM, GAL4DBD-EstR-Msn2TAD; NLS, nuclear localization sequence; Kar2ss, Kar2 signal sequence.

Plasmid	Alias	Source
pFA6a-GFP(S65T)-HIS3	pSS039	Longtine, 1998[1]
pFA6a-mNeonGreen-klTRP1	pSS904	this study
pFA6a-mScarlet-I3-HIS3	pSS1421	this study
pFA6a-mScarlet-I3-klTRP1	pSS1426	this study
pFA6a-sfGFP-klTRP1	pSS1499	this study
pFA6a-sfGFP-HDEL-hph	pSS1544	this study
pFA6a-mCherry-kanMX6	pSS061	this study
pFA6a-nat-P_{CYC}-yeGFP	pSS1066	Janke, 2004[2]
pFA6a-mNeonGreen-HIS3	pSS447	Papagiannidis, 2021[3]
pFA6a-HaloTag-klTRP1	pSS1411	this study
pRS415-P_{TEF}	pSS023	Mumberg, 1995[4]
pRS406-P_{GPD}-mCherry-Ubc6	pSS117	Benoit Kornmann
pRS406-P_{TEF}-mCherry-Ubc6	pSS482	this study
pRS303H-P_{GPD}-TagBFP	pSS349	Szoradi, 2018[5]
pRS406-P_{TEF}-TagBFP-Ubc6	pSS988	this study
pNH605-P_{ADH}-GEM	pDEP151	David Pincus
pFA6a-hph	pSS033	Janke, 2004[2]
pFA6a-kan-GEM-P_{GAL}	pSS669	Szoradi, 2018[5]
pFA6a-nat-P_{GPD}-yeGFP	pSS440	Janke, 2004[2]
pFA6a-hph-P_{GPD}-yeGFP	pSS456	this study
pRS405-P_{GAL}-Kar2ss-sfGFP-HDEL	pSS1223	this study
pFA6a-hph-P_{GAL}-Kar2ss-sfGFP	pSS1367	this study
pNH605-P_{ADH}-GEM-P_{GAL}	pSS474	Schmidt, 2019[6]
pNH605- P_{ADH}-GEM-P_{GAL}-NLS-mNeonGreen-T_{CYC}	pSS1563	this study

1. Longtine, M. S. et al. Additional modules for versatile and economical PCR-based gene deletion and modification in Saccharomyces cerevisiae. *Yeast* **14**, 953-961 (1998).
2. Janke, C. et al. A versatile toolbox for PCR-based tagging of yeast genes: new fluorescent proteins, more markers and promoter substitution cassettes. *Yeast* **21**, 947-962 (2004).
3. Papagiannidis, D. et al. Ice2 promotes ER membrane biogenesis in yeast by inhibiting the conserved lipin phosphatase complex. *EMBO J* **40**, e107958 (2021).
4. Mumberg, D., Müller, R. & Funk, M. Yeast vectors for the controlled expression of heterologous proteins in different genetic backgrounds. *Gene* **156**, 119-122 (1995).
5. Szoradi, T. et al. SHRED Is a Regulatory Cascade that Reprograms Ubr1 Substrate Specificity for Enhanced Protein Quality Control during Stress. *Mol Cell* **70**, 1025-1037 (2018).
6. Schmidt, R. M., Schessner, J. P., Borner, G. H. & Schuck, S. The proteasome biogenesis regulator Rpn4 cooperates with the unfolded protein response to promote ER stress resistance. *Elife* **8**, e43244 (2019).
