# Supplementary material for: Dynamic Organellar Mapping in yeast reveals extensive protein localization changes during ER stress
Source: Nat Commun. 2025 Dec 2;16:10842. doi: 10.1038/s41467-025-66946-8 (PMC12672650; doi:10.1038/s41467-025-66946-8)
Supplement: Supplementary file 14 — Supplementary Data 11 [file 41467_2025_66946_MOESM14_ESM.pdf]

Supplementary Data 11. Yeast strains used in this study. BFP, TagBFP; Cherry, mCherry; GEM, GAL4DBD-EstR-Msn2TAD; Halo, HaloTag; Kar2ss, Kar2 signal sequence; Neon, mNeonGreen; Scarlet, mScarlet-I3; sfGFP, superfolder GFP; yeGFP, yeast-enhanced GFP.

Strain	Relevant genotype	Source
SSY122	*ADE2 leu2-3,112 trp1-1 ura3-1 his3-11,15 MATa*	Szoradi, 2018[1]
SSY4217	*Sec63-Scarlet::HIS3*	this study
SSY4404	*Sec63-Scarlet::HIS3 Pdi1-sfGFP-HDEL::hph*	this study
SSY4845	*Sec63-Scarlet::HIS3 Sil1-sfGFP-HDEL::hph*	this study
SSY4356	*Sec63-Scarlet::HIS3 Ero1-sfGFP::TRP1*	this study
SSY4355	*Rtn1-Cherry::kan* P_{CYC}*-yeGFP-Dpm1::nat Yop1-Halo::TRP1 ura3::*P_{TEF}*-BFP-Ubc6-URA3*	this study
SSY4220	*Sec63-Scarlet::HIS3 Prc1-sfGFP::TRP1*	this study
SSY4279	*Sec63-Scarlet::HIS3 Atg42-sfGFP::TRP1*	this study
SSY4750	*Sec63-Scarlet::HIS3 kan-GEM-*P_{GAL}*-Prc1-sfGFP::TRP1*	this study
SSY4278	*Sec63-Scarlet::HIS3 Pep1-sfGFP::TRP1*	this study
SSY4251	*Sec63-Scarlet::HIS3 leu2::*P_{ADH}*-GEM-LEU2*	this study
SSY4281	*Sec63-Scarlet::HIS3 leu2::*P_{ADH}*-GEM-LEU2* P_{GAL}*-Kar2ss-sfGFP-Gas1::hph*	this study
SSY4847	*Sec63-Scarlet::HIS3 leu2::*P_{ADH}*-GEM-LEU2* P_{GAL}*-Kar2ss-sfGFP-Gas1::hph Pus1-Halo::TRP1*	this study
SSY4280	*Sec63-Scarlet::HIS3 leu2::*P_{ADH}*-GEM-LEU2* P_{GAL}*-Kar2ss-sfGFP-Gas3::hph*	this study
SSY4848	*Sec63-Scarlet::HIS3 leu2::*P_{ADH}*-GEM-LEU2* P_{GAL}*-Kar2ss-sfGFP-Gas3::hph Pus1-Halo::TRP1*	this study
SSY4284	*Sec63-Scarlet::HIS3 leu2::*P_{ADH}*-GEM-LEU2* P_{GAL}*-Kar2ss-sfGFP-Utr2::hph*	this study
SSY4259	*Sec63-Scarlet::HIS3 Mnn2-sfGFP::TRP1*	this study
SSY4260	*Sec63-Scarlet::HIS3 Mnn5-sfGFP::TRP1*	this study
SSY4394	*Sec63-Scarlet::HIS3 Aur1-sfGFP::TRP1*	this study
SSY1212	*ura3::*P_{GPD}*-Cherry-Ubc6-URA3*	this study
SSY3788	*ura3::*P_{GPD}*-Cherry-Ubc6-URA3 Pom152-Neon::HIS3*	this study
SSY3789	*ura3::*P_{GPD}*-Cherry-Ubc6-URA3 Nup170-Neon::HIS3*	this study
SSY3790	*ura3::*P_{GPD}*-Cherry-Ubc6-URA3 Nup159-Neon::HIS3*	this study
SSY3791	*ura3::*P_{GPD}*-Cherry-Ubc6-URA3 Nup133-Neon::HIS3*	this study
SSY3792	*ura3::*P_{GPD}*-Cherry-Ubc6-URA3 Nup57-Neon::HIS3*	this study
SSY3793	*ura3::*P_{GPD}*-Cherry-Ubc6-URA3 Nic96-Neon::HIS3*	this study
SSY3794	*ura3::*P_{GPD}*-Cherry-Ubc6-URA3 Nup1-Neon::HIS3*	this study
SSY3885	*ura3::*P_{GPD}*-Cherry-Ubc6-URA3 Nup82-Neon::HIS3*	this study
SSY3886	*ura3::*P_{GPD}*-Cherry-Ubc6-URA3 Nup116-Neon::HIS3*	this study
SSY3980	*ura3::*P_{GPD}*-Cherry-Ubc6-URA3 Nup49-Neon::HIS3*	this study
SSY3981	*ura3::*P_{GPD}*-Cherry-Ubc6-URA3 Nsp1-Neon::HIS3*	this study
SSY4071	*ura3::*P_{GPD}*-Cherry-Ubc6-URA3 Nup42-Neon::HIS3*	this study
SSY4072	*ura3::*P_{GPD}*-Cherry-Ubc6-URA3 Gle1-Neon::HIS3*	this study
SSY4073	*ura3::*P_{GPD}*-Cherry-Ubc6-URA3 Gle2-Neon::HIS3*	this study
SSY4074	*ura3::*P_{GPD}*-Cherry-Ubc6-URA3 Dyn2-Neon::HIS3*	this study
SSY4129	*ura3::*P_{GPD}*-Cherry-Ubc6-URA3 Nup100-Neon::HIS3*	this study
SSY4410	*ura3::*P_{GPD}*-Cherry-Ubc6-URA3 Nup157-Neon::HIS3*	this study
SSY4411	*ura3::*P_{GPD}*-Cherry-Ubc6-URA3 Nup59-Neon::HIS3*	this study
SSY4413	*ura3::*P_{GPD}*-Cherry-Ubc6-URA3 Ndc1-Neon::HIS3*	this study
SSY4414	*ura3::*P_{GPD}*-Cherry-Ubc6-URA3 Nup53-Neon::HIS3*	this study
SSY4415	*ura3::*P_{GPD}*-Cherry-Ubc6-URA3 Pom34-Neon::HIS3*	this study
SSY4418	*ura3::*P_{GPD}*-Cherry-Ubc6-URA3 Nup188-Neon::HIS3*	this study
SSY4419	*ura3::*P_{GPD}*-Cherry-Ubc6-URA3 Nup84-Neon::HIS3*	this study
SSY4420	*ura3::*P_{GPD}*-Cherry-Ubc6-URA3 Nup120-Neon::HIS3*	this study
SSY4421	*ura3::*P_{GPD}*-Cherry-Ubc6-URA3 Mlp2-Neon::HIS3*	this study

SSY4446	ura3::P_{GPD}-Cherry-Ubc6-URA3 Nup192-Neon::HIS3	this study
SSY4456	ura3::P_{GPD}-Cherry-Ubc6-URA3 Nup85-Neon::HIS3	this study
SSY4457	ura3::P_{GPD}-Cherry-Ubc6-URA3 Nup2-Neon::HIS3	this study
SSY4458	ura3::P_{GPD}-Cherry-Ubc6-URA3 Nup60-Neon::HIS3	this study
SSY4459	ura3::P_{GPD}-Cherry-Ubc6-URA3 Mlp1-Neon::HIS3	this study
SSY4463	ura3::P_{GPD}-Cherry-Ubc6-URA3 Nup145C-Neon::HIS3	this study
SSY4293	ura3::P_{GPD}-Cherry-Ubc6-URA3 Kap95-Neon::HIS3	this study
SSY4812	ura3::P_{GPD}-Cherry-Ubc6-URA3 Nup159-Neon::HIS3 Pus1-Halo::TRP1	this study
SSY4813	ura3::P_{GPD}-Cherry-Ubc6-URA3 Nup157-Neon::HIS3 Pus1-Halo::TRP1	this study
SSY4814	ura3::P_{GPD}-Cherry-Ubc6-URA3 Nup84-Neon::HIS3 Pus1-Halo::TRP1	this study
SSY4815	ura3::P_{GPD}-Cherry-Ubc6-URA3 Nsp1-Neon::HIS3 Pus1-Halo::TRP1	this study
SSY4816	ura3::P_{GPD}-Cherry-Ubc6-URA3 Pom152-Neon::HIS3 Pus1-Halo::TRP1	this study
SSY4817	ura3::P_{GPD}-Cherry-Ubc6-URA3 Nup1116-Neon::HIS3 Pus1-Halo::TRP1	this study
SSY4444	ura3::P_{GPD}-Cherry-Ubc6-URA3 Nup116-Neon::HIS3 Kap95-Halo::TRP1	this study
SSY4571	trp1::P_{TEF}-BFP-Ubc6-TRP1 Nup159-Scarlet::HIS3 Kap95-Halo::kan leu2::P_{ADH}-GEM-P_{GAL}-NLS-Neon-T_{CYC}-LEU2	this study
SSY4574	his3::P_{GPD}-BFP-hph leu2::P_{ADH}-GEM-P_{GAL}-NLS-Neon-T_{CYC}-LEU2 Pus1-Scarlet::TRP1	this study

1. Szoradi, T. et al. SHRED Is a Regulatory Cascade that Reprograms Ubr1 Substrate Specificity for Enhanced Protein Quality Control during Stress. *Mol Cell* **70**, 1025-1037 (2018).
