# Supplementary material for: Dynamic Organellar Mapping in yeast reveals extensive protein localization changes during ER stress
Source: Nat Commun. 2025 Dec 2;16:10842. doi: 10.1038/s41467-025-66946-8 (PMC12672650; doi:10.1038/s41467-025-66946-8)
Supplement: Supplementary file 17 — Supplementary Data 14 [file 41467_2025_66946_MOESM17_ESM.zip › Supplementary Data 14/Instructions for Supplementary Data 14.pdf]

Instructions for Supplementary Data 14

This file contains all data required to recapitulate the DOM-ABC filtering, transformation and analysis of the organellar maps discussed in this study.
There are two separate data folders: one contains the six 'Steady State Maps' (from untreated yeast), the other the nine 'ER Stress Maps' (from untreated control, DTT-treated and tunicamycin-treated cells, each in triplicate). Below is a step-by-step guide to upload and analyse the data.
Please note that the provided protein groups files have been 'trimmed' compared to the original MaxQuant output files, to contain only the data required for DOM-ABC processing. This allows faster data upload.
Most DOM-ABC processing steps typically take a few seconds, but not minutes. If you experience very sluggish response times, try a different web browser. For PCs, we recommend using Firefox or Google Chrome.

1. Analysing the Steady State Maps

Go to
https://domabc.bornerlab.org/QCtool
Click on
Format and analyse single experiment
Now click on **Browse/Choose file** to upload a data file. Within the Supplemental Dataset 3 folder, open the Steady State Maps folder. Select the file
Protein groups Yeast Steady State Maps
Wait until the file has been uploaded - this can take a while. While upload is in progress, the file's name is displayed next to the upload box. When the file name disappears, upload is complete.
Now type this **Experiment name** into the box on the right:
Yeast Steady State Maps
Click somewhere just outside the box to accept the name.
Go to **Column configuration** and click the box **preset patterns**. From the dropdown menu, select the top option (Replicates_Fractions).
Click on **Fractions**. The box will expand to show how DOM-ABC has read the protein groups file data structure based on the preset pattern you selected.
Since we do not want to include the cytosol (Cyt) here, delete 'Cyt' in the **Label** box. Click somewhere just outside the box to confirm the change. Only six fractions remain.
Go to **Protein Annotations** and In the box **Select organism**, choose 'Saccharomyces cerevisiae'. Wait as the page updates, box by box.
In the box **Organelle markers**, pick 'upload custom file', then click on Browse/Choose file and select the file
Yeast markers DOMABC_1908.csv
In the box **Reannotation of gene names**, pick 'from uniprot tab download', wait until the box **Annotation file** appears and upload the file
YeastGenes stream.tab
(This step is not absolutely required, but generates the yeast gene names used in this study.)

In the **Consecutive value filter** box, move the slider to **3**.
Now save the DOM-ABC settings for reference and future use – press the blue **Download domqc_settings.json** button.

(NB: If you want to repeat the analysis, or check how you configured the analysis, you can now re-use the same settings, and skip most of the configuration steps described above: First, upload the protein groups file for the Yeast Steady State Maps as described. Then, restore the saved settings as follows. Next to the blue **Save settings** button at the bottom of the interface is a small grey button called **Load settings** for uploading previous settings. Select the appropriate domqc_settings.json file, and wait for the page to update the configuration boxes one by one. Importantly, the **Organelle markers** and **Reannotation of genes** still have to be specified manually, since they require the upload of external files. A domqc_settings file with saved settings for the Steady State Maps is provided as part of Supplemental Data 14.)

Now press
Run processing
Wait for the analysis to finish.
Scroll down and you can now access the various data tabs for PCA, depth assessment, and download.
For PCA, de-select **Fix aspect ratio by variability**, and **select PC1 vs PC2** to show optimal separation of compartment clusters.
In the download section, you can download the formatted and normalized profiling data as a .csv file, as well as the complete analysis as a .json file. This file is also provided in Supplemental Data 14.

To perform SVM analysis, scroll up to the top and select the
Benchmark
tab. The steady state maps dataset will already be loaded.

(NB: If you want to repeat the analysis at a later stage, you can go directly to the **Benchmark** tab and upload the saved analysed_datasets.json file here. This way you will not have to repeat the 'Format and analyse single experiment' part described above.)

Press
Align and analyse selected datasets
Wait for the analysis to finish.
Scroll down, and select the
SVM analysis
tab.
Select the
Run SVMs
tab.
Set test set proportion to 0.
Press
Run training

The program will iterate through several rounds of training. Once the training has finished, press
Run predictions
When done, select
Download SVM predictions.csv.
A summary of these predictions is provided in Supplementary Figure 1c.

2. Analysing the ER Stress Maps

NB: The following step-by-step guide uses previously saved DOM-ABC configuration settings provided here as part of Supplemental Dataset 3. The protocol for the Steady State Maps (see 1., above) includes a description of how to generate and save configuration settings for future use.

Go to
https://domabc.bornerlab.org/QCtool
Click on
Format and analyse single experiment
Now click on **Browse/Choose file** to upload a data file. From the Supplemental Dataset 3 folder, open the folder 'ER Stress Maps'. Select the file
Protein_groups_ER_stress_all_maps.txt
Scroll down to **Load settings**, and select
domqc_settings_ER_stress_all_maps.json
Wait while the page updates itself, box by box.
In the box **Organelle markers**, upload the custom file
Yeast markers DOMABC_1908.csv
In the box **Annotation file**, upload the custom file
YeastGenes_stream.tab
(This step is not absolutely required, but generates the yeast gene names we used in this study.)
Press
Run processing
Wait for the analysis to finish.
Scroll down and you can now access the various data tabs for PCA, depth assessment, and download.
You can download the profiling data, or the complete analysis as a .json file.
To perform the MR movement analysis, select the tab
Movement analysis
To compare DTT treatment and control maps, press
Calculate/display MR plot
This will take a while.
Scroll down to see the results. These results are also shown in Figure 3c.
To compare tunicamycin treatment and control, select 'Tunicamycin' in the **Condition 2** box near the top of the MR analysis tool, and press
Calculate/display MR plot. Scroll down to see the results. These results are also shown in Figure 3c.

To perform SVMs and cross-dataset benchmarking, you need to repeat the **Format and analyse single experiment** steps above three times, but **for the individual control, DTT and tunicamycin protein groups datasets** provided here. So upload
Protein groups ER stress Control
with
domqc settings ER stress Control
Specify the **Organelle markers** and **Annotation file**; run processing.
Upload
Protein groups ER stress DTT
with
domqc settings ER stress DTT
and run processing.
Upload
protein groups ER stress Tunicamycin
with
domqc settings for ER stress Tunicamycin
and run processing.
Then go to the
Benchmark
tab.
All three individual datasets will already have been loaded. Make sure that only these three datasets are selected, before pressing
Align and analyse selected datasets
Once the analysis is finished, you can access the SVM tool and other functionalities.

SHORTCUT to Benchmarking analysis
Supplemental Dataset 3 also contains the analysed datasets for control, DTT and tunicamycin maps (as .json files). These can be loaded directly into the Benchmark tool. Go to
https://domabc.bornerlab.org/QCtool
Click on
Benchmark multiple experiments
Upload, one by one, the following datasets:

AnalysedDatasets_ER_stress_Control.json
AnalysedDatasets_ER_stress_DTT.json
AnalysedDatasets_ER_stress_Tunicamycin.json

Press
Align and analyse selected datasets
Once the analysis is finished, you can access the SVM tool and other functionalities.

Generating 7-datapoint profiles including the cytosolic fraction

The data upload is as described above – ‘Format and analyse single experiment’, since the protein groups files (‘Steady State Maps’ or ‘ER Stress Maps’) also include the cytosol data. There is only one extra step during setup. Click the **Fractions** button. If you used the saved domqc settings provided here, the **Label** box next to the last fraction will be empty. Now type **Cyt** into this box, to include the cytosolic fraction in the analysis. If you did not use the saved domqc settings, the **Cyt** label should already be there, and no changes are required. Now press **Run processing**. Please note that fraction weighting with protein yields and re-normalization are currently performed outside DOM-ABC (see Methods for details).
